# Growth, Gastrointestinal Tolerance and Stool Characteristics of Healthy Term Infants Fed an Infant Formula Containing Hydrolyzed Whey Protein (63%) and Intact Casein (37%): A Randomized Clinical Trial

**DOI:** 10.3390/nu9111254

**Published:** 2017-11-16

**Authors:** Shang-Ling Wu, Ding Ding, Ai-Ping Fang, Pei-Yan Chen, Si Chen, Li-Peng Jing, Yu-Ming Chen, Hui-Lian Zhu

**Affiliations:** 1 Department of Nutrition, School of Public Health, Sun Yat-sen University, Guangzhou 510080, China; wushling3@mail2.sysu.edu.cn (S.-L.W.); fangaip@mail.sysu.edu.cn (A.-P.F.); chpyan@mail2.sysu.edu.cn (P.-Y.C.); chens37@mail2.sysu.edu.cn (S.C.); 2 Department of Statistics and Epidemiology, School of Public Health, Sun Yat-sen University, Guangzhou 510080, China; dingd3@mail2.sysu.edu.cn (D.D.); jinglp@mail2.sysu.edu.cn (L.-P.J.); chenyum@mail.sysu.edu.cn (Y.-M.C.)

**Keywords:** hydrolyzed whey protein, infant formulas, growth, gastrointestinal tolerance, stool characteristics, healthy term infants

## Abstract

To investigate whether healthy term infants, fed an infant formula containing hydrolyzed whey protein (HWP-F, hydrolyzed whey/intact casein =63/37), differ in growth, gastrointestinal tolerance and stool characteristics from those fed an infant formula containing intact whey protein (IWP-F, intact whey/intact casein =61/39) or breast milk. Healthy term infants, born within 14 days of the study’s commencement, were randomly assigned to be fed IWP-F or HWP-F until 13 weeks of age, and breast-fed (BF) infants were enrolled as a reference group. Anthropometric measurements, gastrointestinal tolerance indexes and stool characteristics were assessed at baseline, and 7 and 13 weeks of age. There were no significant differences in any growth measurements and the occurrence of crying, spit-up and difficult defecation among the three feeding groups during the study period. However, daily feeding frequency was consistently lower in the formula-fed infants than in the BF group throughout the study (*p* < 0.05), and infants in the HWP-F group consumed more formula than those in the IWP-F group at 7 and 13 weeks of age (*p* ≤ 0.002). The HWP-F-fed infants had more similar stool characteristics to the breast-fed infants than infants in the IWP-F group at 13 weeks of age, regardless of frequency, volume, color or consistency of stool. This study demonstrates that the HWP-F could support the normal growth of healthy term infants, to a comparable extent to that of breast-fed infants during the first three months of life. Moreover, stool characteristics of HWP-F-fed infants are much closer to breast-fed infants than IWP-F-fed infants, but no significant gastrointestinal tolerance improvement was observed in HWP-F group.

## 1. Introduction

Breast milk provides unique benefits to both infants and mothers; however, only 37% of infants younger than 6 months are exclusively breastfed [1,2]. Infant formulas are designed to be a substitute for breast milk for infants who cannot be breast-fed because of metabolic diseases, maternal infectious diseases or maternal breast insufficiency [3]. Infants experience rapid growth and development and are sensitive to nutritional adequacy during the early postnatal period. However, the difference in formula processing—e.g., protein source and the degree of hydrolysis—may affect the bioavailability and stability of nutrients, and thus result in different growth patterns. Traditional formula, referring to the cow-milk based intact protein formula which is applied most extensively, occupies 83% of market share [4]. However, infants with early exposure to cow-milk intact protein formulas are more likely to develop a cow milk protein allergy [5] and gastrointestinal intolerance symptoms [6,7].

Hydrolyzed protein formulas have been developed for infants at a high risk for cow milk protein allergy and preterm infants who are intolerable to intact cow-milk based standard infant formulas, when breastfeeding is not available. More recently, there has been growing evidence that preterm infants fed hydrolyzed infant formulas have a better feeding tolerance, as indicated by higher gastrointestinal transport rates, more rapid gastric emptying, less gastro-esophageal reflux, and more rapid establishment of full enteral feeding [8,9,10,11,12,13]. As a result, replacing intact protein with hydrolyzed protein in infant formulas might also have beneficial effects on improving feeding tolerance in healthy term infants, especially during the early postnatal period [14,15].

Up until now, few clinical trials have been conducted to examine the potentially beneficial effects of hydrolyzed infant formulas on growth and gastrointestinal tolerance in healthy term infants. The objective of present study was to evaluate the growth, gastrointestinal tolerance and stool characteristics of healthy term infants fed an infant formula containing hydrolyzed whey protein, during the first 3 months of life.

## 2. Materials and Methods 

### 2.1. Study Design

This study was a randomized, controlled, parallel, multicenter, clinical trial, conducted in healthy, full-term infants during the first 3 months of life. Infants, whose mothers had elected to formula feed, were recruited by medical staff, between 7 December 2015 and 28 July 2016, from obstetric wards of general and specialized hospitals in Shaoyang (*n* = 156), Nanxiong (*n* = 5), and Zhengzhou (*n* = 28), China. Formula-fed infants, aged ≤ 14 days were randomized to one of two study formulas: the formula containing hydrolyzed whey protein (HWP-F) or intact whey protein (IWP-F). Infants were exclusively fed their assigned formula from enrollment until 13 weeks of age. In addition, a non-randomized reference group of breast-fed infants was enrolled and followed. The trial was registered at http://www.clinicaltrials.gov (ClinicalTrials.gov Identifier NCT03178474). The study was conducted according to the guidelines laid down in the Declaration of Helsinki and all procedures involving human subjects were approved by the Ethical Review Board at School of Public Health, Sun Yat-Sen University (No. 2015-48, approved on 13 October 2015) and informed consent was obtained from each mother before study entry.

### 2.2. Subjects

Eligibility for study participation required infants to be healthy, full-term infants, aged ≤ 14 days, with a gestational age between 37 and 42 weeks, a birth weight ranging from 2500 to 4000 g, Apgar Scores above 7 at 5 and 10 min after birth, and negative for Hepatitis B surface antigens. Infants with a suspected or known allergy to cow’s milk protein and those with any maternal, fetal or perinatal history that could adversely affect feeding and growth were excluded from all groups, and infants who were fed breast milk for more than 2 weeks were excluded from the formula-fed groups.

### 2.3. Study Formulas

The infant formulas were commercially available and were almost identical, apart from the degree of whey protein hydrolysis. The control formula (Infant formula containing intact whey protein, IWP-F; Yili^®^, Hohhot, China) provided 0.90 g of intact whey protein and 0.58 g intact casein protein per 100 mL (whey:casein = 61:39), while the experimental formula (Infant formula containing hydrolyzed whey protein, HWP-F; Yili^®^, Hohhot, China) supplied 0.97 g of hydrolyzed whey protein and 0.58 g intact casein protein per 100 mL (whey:casein = 63:37). In the HWP-F, 19.6% of peptides had a molecular weight below 2000 Da; 7.9% were between 2000~5000 Da and 72.5% were above 5000 Da (Supplementary Materials Table S1). In addition, both the formulas had added lactose, docosahexaenoic acid (DHA) and arachidonic acid (ARA), supplied by the Inner Mongolia Yili Industrial Group Co., Ltd., Dongguan, China. The nutrient compositions of the study formulas are shown in Table 1.

### 2.4. Study Procedures

Randomization to the study formulas was achieved by using computer-generated random numbers. Allocation concealment was performed by putting randomization numbers in sealed, sequentially numbered envelopes. Formulas were supplied as powders and parents were instructed to feed the assigned study formula ad libitum to infants as the sole source of nutrition during the study. Study visits were scheduled when infants were 2, 7 and 13 weeks of age.

Demographic information of all infants (e.g., gender, age, gestational age at birth, birth weight, and delivery mode) and their mothers (e.g., age, height, weight, education level, smoking status during pregnancy, and contact information) were collected by using a face-to-face questionnaire at enrollment.

Body weight, crown–heel length, and head circumference of infants were measured by trained personnel at enrollment, 7 and 13 weeks of age. Infants were weighed in light clothing and diapers on a calibrated electronic scale to the nearest 10 g (Intelligent physical examination instrument, Wuruan^®^, Wuhan, China). Crown–heel length was measured by using an infant horizontal measurement bed, with the infant in a supine position, to the nearest 0.1 cm. Head circumference was measured at the largest occipital frontal circumference, to the nearest 0.1 cm, with a flexible rule. Each measurement was taken twice, and the average was used for the analysis. A third measurement was made if the difference of the first two measurements in weight was greater than 50 g, or if the difference in crown–heel length and head circumference was greater than 1 cm. Infant body mass index (BMI) was calculated as weight in kilograms divided by crown–heel length in meters squared. Gender-specific weight-for-age *z*-scores, length-for-age *z*-scores, head circumference-for-age *z*-scores, BMI-for-age *z*-scores, and weight-for-length *z*-scores of infants were calculated based on the World Health Organization (WHO) child growth standards (version 3.2.2, January 2011) [16]. Gains in weight, length and head circumference from baseline to 7 or 13 weeks of age were calculated as the differences divided by the exact number of elapsed days between measurements.

Three-day intake and stool diaries were completed by parents before the 2, 7, and 13 week visits. These records included infant feeding (e.g., number of daily feeding, and daily volume of formula intake), gastrointestinal tolerance (e.g., occurrence of crying, constipation, spit-up, and adverse events), and stool characteristics (i.e., stool frequency, volume, color and consistency). Adverse events were coded using the WHO Adverse Reactions Terminology (WHO-ART). Infant difficult defecation was deemed as the passage of large-diameter stools with retentive posturing and avoiding defecation by purposefully contracting the pelvic floor. The volume scores per stool were expressed as stool diameter, on a scale of 1 to 3 (1 ≤ 7.5 cm, 2 > 7.5 cm and < 13.0 cm, 3 ≥ 13.0 cm). Stool color score was evaluated by the Infant Stool Color Chart [17] on a scale of 1 to 9 (1 = most abnormal to 9 = most normal). Stool consistency score was assessed by the simplified Bristol Stool Form Scale [18], and each stool was ranked by parents on a scale of 1 to 6 (1 = hard lumps, 2 = sausage-shaped but lumpy, 3 = formed but with cracks, 4 = smooth and soft, 5 = soft blobs with clear-cut edges, 6 = mushy). The stool ranks were averaged for each day, and then these daily means were averaged over the study period. Information recorded in the diaries was reviewed and verified by researchers during each study visit.

Compliance was assessed through questionnaire interviews at each visit and through telephone interviews between the visits. Noncompliant infants, who were fed the study formulas for less than 80% of feedings in the intervention groups, or who were fed infant formulas exceeding 20% of feedings in the breast-fed group during the study period, were excluded from further study participation. Infants were considered lost to follow-up if their parents could not be contacted or traced any more, or refused further participation.

### 2.5. Sample Size 

We considered weight gain as the primary outcome. Calculation of sample size was based on weight gain data during the first 3 months of life, as recommended by the American Academy of Pediatrics [19]. A sample size of 48 infants was needed in each formula group, using a 3 g/day difference in weight gain, with a standard deviation (SD) of 4.5 g/day, to ensure a power of 90% and a type I error of 5%. To compensate for an expected 20% dropout rate, enrollment of 60 infants per group was thus, planned.

### 2.6. Statistical Analysis

We conducted both intention-to-treat (ITT) analyses and per protocol (PP) analyses. The former included all of the randomized infants with available data, while the latter only included compliant infants. Only ITT data are presented. Continuous variables were reported as means ± SDs or medians and interquartiles (IQRs), and categorical variables as counts and percentages. We performed an analysis of variance (ANOVA) for normally-distributed data, Kruskal-Wallis H rank-sum tests for skewed distributed data, and chi-square tests for categorical data, to compare differences among the three groups. If an overall test was statistically significant, a post-hoc analysis was conducted by using the Bonferroni test, to compare differences between any two groups. Anthropometric measurements and stool characteristics were compared using repeated measures ANOVA, with treatment, time, and treatment*time as factors. Gains in weight, length and head circumference were compared using ANOVA and analysis of covariance (ANCOVA). Potential confounders included infant birth weight, gender, recruited center, baseline weight, length and head circumference for adjustment. All tests were two-sided, and a significant difference was set as a *p* value less than 0.05 for all analyses. Data were analyzed by using the SPSS version 20 for Windows package (IBM Statistics, IBM Corporation, New York, NY, USA).

## 3. Results

### 3.1. Subject Characteristics 

**Figure 1:**
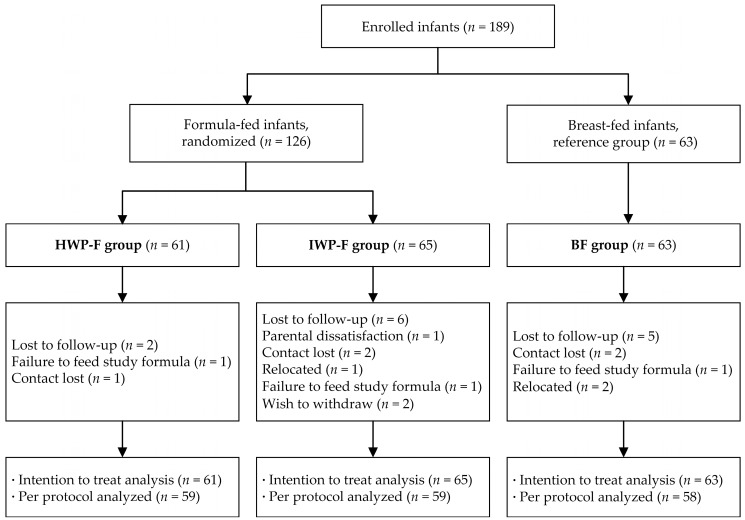
Flow of the study design. Note: HWP-F, Infant formula containing hydrolyzed whey protein; IWP-F, Infant formula containing intact whey protein; BF, breast-fed.

One hundred and eighty-nine infants were enrolled into the study, with 63 in the breast-fed (BF) group, 65 in the IWP-F group and 61 in the HWP-F group. A total of 13 infants withdrew during the study period, which yielded final group sizes of 59 for both the IWP-F and HWP-F groups and 58 for the BF group (Figure 1). There were no significant differences among the three groups in dropout rates (*p* = 0.445). Baseline characteristics were similar in the three groups, except for infant gender (Table 2).

### 3.2. Growth

There were neither significant independent group effects, nor significant group-by-time interaction effects, on any growth measurements (Table 3). Similarly, no significant differences were observed for gains in weight, length, and head circumference in the three groups from entry to 7 or 13 weeks of age, whether adjusting for birth weight, gender, and baseline growth indices of infants, or not (Table 4).

### 3.3. Gastrointestinal Tolerance

Details about the intake and tolerance to study formulas are presented in Table 5. The amount of daily feeding was not significantly different between the formula-fed groups, but both had less feeding than the BF group during the study period. There was a consistently greater (~20%) intake volume of HWP-F than IWP-F at 7 and 13 weeks of age, but no group-by-time interaction effect (*p* = 0.116) was seen.

There were no significant differences in the amount of daily crying and the occurrence of adverse events, difficult defecation, and spit-up in infants fed HWP-F, IWP-F and BF during the first 13 weeks of life.

### 3.4. Stool Characteristics 

**Figure 2:**
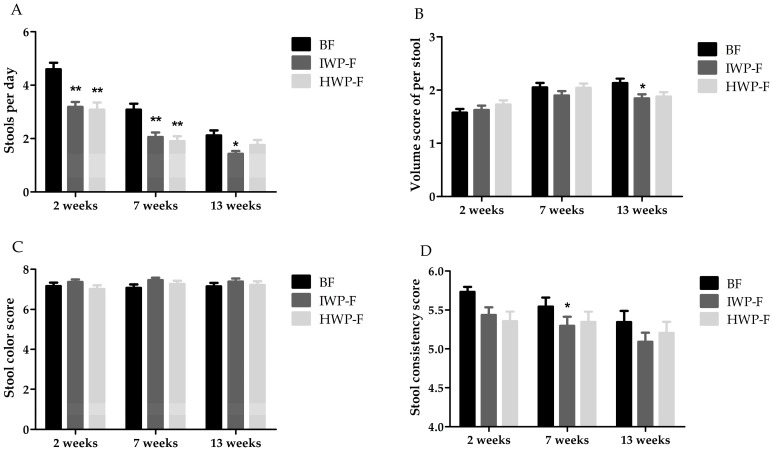
Stool characteristics (shown as mean and standard error) at 2, 7 and 13 weeks of age in breast-, IWP-F-, and HWP-F-fed term infants. (**A**) Stool frequency, indicated as stools per day; (**B**) Volume score per stool, expressed as stool diameter: 1 ≤7.5 cm; 2 >7.5 cm and <13.0 cm; 3 ≥13.0 cm; (**C**) Stool color score, calculated using the Infants Stool Color Chart, 1 indicates the most abnormal stool score, and 9 is the most normal; (**D**) Stool consistency score, rated based on the simplified Bristol Stool Form Chart, 1 = hard lumps, 2 = sausage-shaped but lumpy, 3 = formed but with cracks, 4 = smooth and soft, 5 = soft blobs with clear-cut edges, 6 = mushy. Thus, the range of possible scores is 0–6; a higher score is closer to a softer stool. Significant differences compared with the BF group are indicated as follows: **: *p* < 0.001; *: *p* < 0.05. HWP-F, Infant formula containing hydrolyzed whey protein; IWP-F, Infant formula containing intact whey protein; BF, breast-fed.

At 2 and 7 weeks of age, formula-fed infants had significantly fewer stools per day than breast-fed infants; however, there were no significant differences in stool frequency between the formula-fed groups (Figure 2A). Similar stool volume and color were observed in the HWP-F and BF groups across the study (Figure 2B,C). Formula-fed infants had lower stool consistency scores than breast-fed infants at 2 weeks of age; however, the significant difference in stool hardness only appeared between the IWP-F and BF groups at 7 weeks of age (Figure 2D). Stool patterns were similar between infants fed HWP-F and breast milk at 13 weeks of age; however, infants fed IWP-F had significantly fewer and smaller stools compared with infants in the other two groups. Decreasing trends in stool frequency and consistency were observed in the three groups throughout the study (*p* < 0.005).

## 4. Discussion

Our study demonstrated that the growth of healthy, full-term infants, fed an infant formula containing hydrolyzed whey protein, during the first 3 months of life, was similar to that of those fed an infant formula containing intact whey protein or breast milk. Moreover, stool characteristics of HWP-F-fed infants were much closer to those of breast-fed infants, but no significant gastrointestinal tolerance improvement was observed in HWP-F-fed infants, compared with IWP-F-fed and breast- fed infants.

The primary results for growth variables were consistent with previous studies. Hernell et al. [20] reported that growth indices, including weight and length, from 6 weeks to 6 months, had no significant differences in infants fed a regular milk formula, a casein-hydrolysate formula, a whey-hydrolysate formula, or breast milk. Regurgitating infants, fed a formula based on whey hydrolysate for a 1-month period, had similar weight gains to those fed a non-hydrolyzed protein formula, and regurgitation times and volume decreased significantly in the hydrolyzed formula fed group [21]. A study by Florendo et al. [22] showed that preterm infants, fed a non-hydrolyzed formula or a partially hydrolyzed whey formula, both had normal gains in weight, length and head circumference, and the infants’ mean daily intakes of formula in the two groups were not statistically different. Julie et al. [23], however, analyzed *z*-score trajectories across infants aged 2.5 to 7.5 months and found that weight gain was accelerated in the cow-milk-formula-fed health infants, whereas weight gain in the hydrolyzed-formula-fed infants was normative. Moreover, the German Infant Nutritional Intervention Study (GINI) also found that weight gain, length gain and BMI of infants with atopic heredity, who were fed a partially hydrolyzed whey formula (pHF-W), an extensively hydrolyzed whey formula, a cow-milk formula or breast milk were no significantly different during the first 10 years of life, but feeding with extensively hydrolyzed casein (eHF-C) formula led to a transient lower weight gain in the first year of life [24,25]. In our study, the HWP-F formula, containing hydrolyzed whey protein with 19.6% of peptides less than 2000 Da, and 72.5% of peptides greater than 5000 Da, and the degree of whey protein hydrolysis was much lower than that of commercially pHF-W and eHF-C, manufactured by Nestlé and Mead Johnson (Supplementary Materials Figure S1) [26], which could provide safe and adequate amounts of nutrients to keep healthy term infants growing normally during the first three months of life.

Daily average numbers of feedings in formula-fed groups were both less than the BF group during the study period, though there were no significant differences between the two formula-fed groups, which might be due to the higher content of protein in formulas [27]. HWP-F-fed infants consumed larger volumes of formula than IWP-F-fed infants, according to feeding diaries. However, the findings were inconsistent with previous studies, based on laboratory measures [23] or diaries [28], which found that healthy term infants, randomly assigned to be fed with HWP-F, had lower intakes than those assigned to fed IWP-F. These authors considered that subjective factors, such as taste, might have been partly responsible for this result, i.e., infants found HWP-F highly pleasant and acceptable throughout the study period. Miraglia et al. [29] considered that whey-protein-hydrolyzed formulas had better palatability than casein-protein-hydrolyzed formulas and amino acid-based formulas. Moreover, introducing HWP-F to infants in the first 3 months of life contributed to improving infant acceptability [30]. However, as observed in the previous studies [31,32], the HWP-F-fed infants consumed significantly larger volumes of milk, but showed relatively slower weight gains than the IWP-P-fed infants, suggesting that HWP-F might have diminished nutrient utilization. Further studies are needed to address the potential mechanisms.

The gastrointestinal tolerance was also evaluated in this study as one of the main outcomes. The results suggested that HWP-F was well tolerated and was possibly better accepted by healthy term infants than IWP-F. However, there were no significant differences in the occurrences of crying, spit-up and difficult defecation among the three groups, contrary to previous studies [21,33], which may be influenced by the degree of hydrolysis and the type of protein. Although functional gastrointestinal disorders, such as spit-up and constipation, were considered benign conditions, and occurred in up to 50% of all infants, each accounting for 15–25% [15,34], these symptoms made parents feel anxious and some even asked for medical consultations.

The stool characteristics data indicated that HWP-F-fed infants were much closer to breast-fed infants than IWP-F-fed infants, especially in the end of study. The current study is one of only two studies to demonstrate stool characteristics of healthy infants fed formula containing hydrolyzed whey protein. In a 3-month feeding study, Schmelzle et al. [28] reported that healthy term infants, fed an infant formula containing partially hydrolyzed protein, had softer stools than infants fed standard formula, which were consistent with the findings of our study, but our study further assessed stool frequency and volume. In another crossover trial, constipated infants, fed an infant formula with added sn-2 palmitate, prebiotic galacto-oligosaccharides and fructo-oligosaccharides and partially hydrolyzed whey protein, had softer stools than those fed standard formulas [6]. Therefore, the results in our study indicated that HWP-F had positive effect on stool characteristics during the first 3 months of infancy. Stool consistency was correlated to whole gut transit time [35], and hydrolyzed protein was an efficient marker for accelerating the intestinal transit time via increasing the intraluminal osmotic load, inducing higher motilin levels and reducing the activity of milk protein-derived opioid receptor agonists [9,10,11,12,13].

No significant differences were seen among the three groups for the occurrence of advert events—e.g., cold, diarrhea, bronchitis, pneumonia—and no fatal diseases happened during the study period. Moreover, the per protocol (PP) analysis, excluding withdrawn infants (*n* = 13) and those who fell outside the tolerance limit in the two visits (*n* = 5) was conducted and no substantial differences in all variables were found, and a stratification analysis by gender and a sensitivity analysis confined to 156 infants (82.5%) in Shaoyang also did not change our conclusion.

This study is the first randomized trial in China to explore the effect of an infant formula containing hydrolyzed whey protein on gastrointestinal tolerance and stool characteristics in health term infants during the first three months of life. There were also some limitations in this study. This was an open label trial and the allocation sequence was available to the research staff and parents throughout the study. Despite this, growth measurements were measured by trained personnel who were blind to the formula codes. Thus, ascertainment bias of growth parameters was unlikely to influence the results. In addition, regarding ethical consideration, the breast-fed group was a non-randomized reference group, and a selection bias between the formula-fed and breast-fed groups cannot be ruled out. Furthermore, a 3 month follow-up could not reflect the long-term effects of HWP-F. Further studies with longer intervention periods and larger sample sizes are needed.

## 5. Conclusions

In conclusion, this study demonstrates that HWP-F supports the normal growth of healthy term infants, to an extent comparable to that of breast-fed infants, during the first three months of life. Moreover, stool characteristics of HWP-F-fed infants were much closer to breast-fed infants than IWP-F-fed infants, but no significant gastrointestinal tolerance improvement was observed in the HWP-F group.

## Figures and Tables

**Table 1:** Composition of study formulas.

	IWP-F (per 100 mL)	HWP-F (per 100 mL)
Energy, kJ	285	287
Protein, g	1.47	1.55
Intact whey protein, g	0.90	/
Hydrolyzed whey protein, g	/	0.97
intact casein, g	0.57	0.58
Whey/casein	61:39	63:37
Fat, g	3.74	3.73
Linoleic acid, g	0.57	0.57
ɑ-linolenic acid, mg	57	57
DHA, mg	7.5	7.5
ARA, mg	10.6	10.8
Carbohydrate, g	7.0	7.0
Lactose, g	6.6	6.6
GOS, FOS, g	0.4	0.4
Vitamins (vits)		
Vitamin A, μgRE	51	52
Vitamin D, μg	1.12	1.14
Vitamin E, mg α-TE	0.92	0.81
Vitamin K1, μg	9.2	8.1
Vitamin B1, μg	73	70
Vitamin B2, μg	92	88
Vitamin B6, μg	55.4	51.3
Vitamin B12, μg	0.198	0.203
Vitamin C, mg	7.9	7.4
Minerals		
Calcium, mg	46	41
Phosphorus, mg	29	27
Magnesium, mg	4.8	5.4
Sodium, mg	17	18
Kalium, mg	49	43
Chlorine, mg	42	51
Iron, mg	0.63	0.63
Zinc, mg	0.50	0.50
Copper, μg	46.2	47.3
Iodine, μg	7.7	8.3
Manganese, μg	4.0	4.4
Selenium, μg	1.91	1.73

IWP-F, Infant formula containing intact whey protein; HWP-F, Infant formula containing hydrolyzed whey protein; DHA, docosahexaenoic acid; ARA, arachidonic acid; GOS, galacto-oligosaccharides; FOS, fructo-oligosaccharides.

**Table 2:** Baseline characteristics according to formula group.

	Group			*p*
	BF (*n* = 63)	IWP-F (*n* = 65)	HWP-F (*n* = 61)	
**Infant characteristics**				
Gender, male, *n* (%)	29 (46.0)	29 (44.6)	41 (67.2) ^1^	0.019
Age at study entry, days, median(IQR)	5.0 (3.0,7.0)	6.0 (3.0,9.0)	6.0 (2.0,9.0)	0.236
Birth weight, kg, mean ± SD	3.4 ± 0.4	3.4 ± 0.4	3.4 ± 0.3	0.532
Gestational age at birth, week, mean ± SD	39.2 ± 1.1	39.6 ± 1.1	39.4 ± 1.0	0.252
Caesarean delivery, *n* (%)	34 (54.8)	39 (60.0)	32 (52.5)	0.682
**Maternal characteristics**				
Age at study entry, years, mean ± SD	28.5 ± 4.1	29.1 ± 4.7	28.4 ± 4.7	0.687
BMI at study entry, kg/m^2^, median(IQR)	22.8 (19.6,27.9)	23.3 (21.5,25.8)	23.4 (21.3,26.1)	0.396
Parity, primiparous, *n* (%)	21 (33.3)	27 (42.2)	25 (41.0)	0.556
**Education level, *n* (%)**				
Primary school or below	10 (15.9)	18 (27.7)	16 (26.2)	0.434
Secondary school	20 (31.7)	22 (33.8)	19 (31.1)	
High school or above	33 (52.4)	25 (38.5)	26 (42.6)	
Active or passive smoking during pregnancy, *n* (%)	13 (20.6)	20 (30.8)	16 (26.2)	0.424

BF, breast-fed; IWP-F, Infant formula containing intact whey protein; HWP-F, Infant formula containing hydrolyzed whey protein; SD, standard deviation; IQR, interquartile range. ^1^ Significantly different from BF (*p* = 0.017) and IWP-F (*p* = 0.011) groups.

**Table 3:** Weight, length, head circumference, and BMI and their *z*-scores at baseline, and 7 and 13 weeks of age, of breast-, IWP-F-, and HWP-F-fed term infants.

	Baseline			*p* ^3^	7 Weeks			*p* ^3^	13 Weeks			*p* ^3^	*p* ^4^
	BF (*n* = 63)	IWP-F (*n* = 65)	HWP-F (*n* = 61)		BF (*n* = 63)	IWP-F (*n* = 65)	HWP-F (*n* = 61)		BF (*n* = 63)	IWP-F (*n* = 65)	HWP-F (*n* = 61)		
**Absolute values** ^1^													
Weight, kg	3.6 ± 0.5	3.5 ± 0.4	3.6 ± 0.5	0.708	5.3 ± 0.5	5.3 ± 0.5	5.2 ± 0.7	0.759	6.5 ± 0.6	6.5 ± 0.6	6.5 ± 0.8	0.927	0.379
Crown–heel length, cm	51.3 ± 2.1	51.3 ± 2.0	51.4 ± 2.2	0.506	57.4 ± 2.0	57.5 ± 2.1	57.6 ± 2.3	0.893	61.5 ± 2.0	61.7 ± 2.1	61.6 ± 2.7	0.855	0.857
Head circumference, cm	34.9 ± 1.4	34.8 ± 1.2	35.1 ± 1.3	0.605	38.1 ± 1.2	37.7 ± 1.1	38.1 ± 1.3	0.099	39.9 ± 1.1	39.8 ± 1.1	39.9 ± 1.2	0.915	0.405
BMI, kg/m^2^	13.5 ± 1.3	13.3 ± 1.2	13.4 ± 1.4	0.830	16.0 ± 1.3	15.9 ± 1.2	15.7 ± 1.6	0.345	17.1 ± 1.5	17.1 ± 1.4	17.1 ± 1.6	0.995	0.543
***z*-scores** ^1,2^													
Weight-for age *z*-score	0.22 ± 0.87	0.21 ± 0.76	0.29 ± 0.96	0.852	0.33 ± 0.70	0.40 ± 0.73	0.11 ± 1.03	0.129	0.43 ± 0.77	0.50 ± 0.65	0.31 ± 0.91	0.393	0.157
Length-for-age *z*-score	0.30 ± 1.08	0.39 ± 1.09	0.52 ± 1.24	0.551	0.36 ± 0.94	0.53 ± 0.97	0.37 ± 1.04	0.543	0.41 ± 0.92	0.52 ± 1.02	0.29 ± 1.21	0.480	0.401
Weight-for-length *z*-score	−0.32 ± 1.18	−0.43 ± 1.17	−0.42 ± 1.20	0.832	0.12 ± 1.05	0.02 ± 1.01	−0.11 ± 1.11	0.482	0.25 ± 1.02	0.25 ± 0.95	0.22 ± 1.23	0.983	0.886
Head circumference-for-age *z*-score	0.06 ± 1.11	0.03 ± 0.98	0.15 ± 1.05	0.798	−0.04 ± 0.95	−0.30 ± 0.93	−0.20 ± 1.05	0.345	−0.11 ± 0.84	−0.16 ± 0.88	−0.31 ± 0.96	0.460	0.302
BMI-for-age *z*-score	0.06 ± 1.01	−0.03 ± 0.89	−0.01 ± 1.07	0.871	0.18 ± 0.89	0.15 ± 0.83	−0.05 ± 1.00	0.309	0.28 ± 0.96	0.29 ± 0.85	0.20 ± 1.11	0.861	0.740

BF, breast-fed; IWP-F, Infant formula containing intact whey protein; HWP-F, Infant formula containing hydrolyzed whey protein; BMI, body mass index; SD, standard deviation; IQR, interquartile range. ^1^ Values are means ± SDs; ^2^ *z*-scores were calculated by using the World Health Organisation (WHO) growth standards; ^3^ The *p* values were derived from one-way analysis of variance (ANOVA) model tests for the difference between the three groups; ^4^ The *p* values represent the time × treatment interactions, derived from repeated measures ANOVAs.

**Table 4:** Gains in weight, length, and head circumference of study infants.

	Group			*p* ^2^	*p* ^3^
	BF (*n* = 63)	IWP-F (*n* = 65)	HWP-F (*n* = 61)		
**Weight, g/day ^1^**					
Baseline–7 weeks	40.5 ± 10.3	42.1 ± 11.4	38.2 ± 13.7	0.195	0.079
Baseline–13 weeks	34.6 ± 6.4	35.3 ± 7.0	34.1 ± 8.4	0.618	0.325
**Crown–heel length, cm/day ^1^**					
Baseline–7 weeks	0.14 ± 0.05	0.15 ± 0.05	0.14 ± 0.05	0.268	0.119
Baseline–13 weeks	0.12 ± 0.02	0.12 ± 0.03	0.12 ± 0.03	0.343	0.313
**Head circumference, cm/day ^1^**					
Baseline–7 weeks	0.07 ± 0.03	0.07 ± 0.03	0.07 ± 0.03	0.535	0.396
Baseline–13 weeks	0.06 ± 0.01	0.06 ± 0.02	0.06 ± 0.02	0.568	0.566

BF, breast-fed; IWP-F, Infant formula containing intact whey protein; HWP-F, Infant formula containing hydrolyzed whey protein; ^1^ Values are means ± SDs; ^2^ The *p* values were derived from one-way analyses of variance; ^3^ The *p* values were derived from covariance analyses, adjusting for infant birth weight, gender, recruited center and growth indices at baseline.

**Table 5:** Feeding tolerance of study infants.

	Group			*p* ^2^
	BF (*n* = 63)	IWP-F (*n* = 65)	HWP-F (*n* = 61)	
**Number of daily feedings ^1^**				
2 weeks	9.7 ± 1.8	8.7 ± 1.7 ^3^	8.6 ± 1.8 ^3^	0.002
7 weeks	9.4 ± 1.7	8.4 ± 1.7 ^3^	8.4 ± 1.6 ^3^	0.002
13 weeks	8.7 ± 1.7	7.7 ± 1.6 ^3^	7.8 ± 1.6 ^3^	0.002
**Volume of formula intake, mL/kg/day ^1^**				
2 weeks	N/A	128.8 ± 47.8	135.3 ± 52.2	0.445
7 weeks	N/A	127.9 ± 37.4	153.4 ± 51.7	0.002
13 weeks	N/A	117.7 ± 34.1	140.9 ± 39.3	0.001
**Number of daily crying ^1^**				
2 weeks	1.3 ± 2.2	1.2 ± 2.1	1.1 ± 1.7	0.905
7 weeks	0.9 ± 1.9	1.3 ± 2.2	1.0 ± 1.8	0.441
13 weeks	0.4 ± 1.2	0.8 ± 1.9	0.7 ± 1.5	0.385
**Episodes of adverse events, *n* (%)**				
Baseline–7 weeks	2 (3.2)	5 (7.7)	2 (3.3)	0.515
7 weeks–13 weeks	7 (11.1)	8 (12.3)	5 (8.2)	0.745
**Episodes of difficult defecation, *n* (%)**				
Baseline–7 weeks	5 (7.9)	13 (20.0)	11 (18.0)	0.130
7 weeks–13 weeks	7 (11.1)	12 (18.5)	6 (9.8)	0.300
**Episodes of spit-up, *n* (%)**				
Baseline–7 weeks	21 (33.3)	28 (43.1)	18 (29.5)	0.257
7 weeks–13 weeks	19 (30.2)	24 (36.9)	17 (27.9)	0.522

BF, breastfeeding; IWP-F, Infant formula containing intact whey protein; HWP-F, Infant formula containing hydrolyzed whey protein; N/A, not available; ^1^ Values are expressed as means ± SDs; ^2^ *p* values were derived from ANOVAs for normally-distributed data, and chi-square tests or Fisher’s exact tests for categorical data; ^3^ Pairwise comparison was done using the Bonferroni test, A significantly difference from the BF group was indicated by *p* < 0.05.

## Supplementary Materials

The following are available online at www.mdpi.com/2072-6643/9/11/1254/s1. Figure S1. Comparation of Molecular weight distribution between HWP-F and commercial pHF-W and eHF-C. HWP-F, Infant formula containing hydrolyzed whey protein. pHF-W and eHF-C are well-established products [1], Table S1: Molecular weight distribution in HWP-F, Table S2. Weight, length, head circumference, and BMI and their z-scores at baseline, and 7 and 13 weeks of age of breast-, IWP-F-, and HWP-F-fed term infants (Per protocol analyzed), Table S3. Gains in weight, length, and head circumference of study infants (Per protocol analyzed), Table S4. Feeding tolerance of study infants (Per protocol analyzed).
