# Supplementary material for: Growth, Gastrointestinal Tolerance and Stool Characteristics of Healthy Term Infants Fed an Infant Formula Containing Hydrolyzed Whey Protein (63%) and Intact Casein (37%): A Randomized Clinical Trial
Source: Nutrients. 2017 Nov 16;9(11):1254. doi: 10.3390/nu9111254 (PMC5707726; doi:10.3390/nu9111254)
Supplement: Supplementary file 1 [file nutrients-09-01254-s001.pdf]

Supplementary materials:

Table S1. Molecular weight distribution in HWP-F

Molecular weight range, Da	HWP-F, %
<180	3.69
180~500	5.55
500~1,000	3.85
1,000~2,000	6.49
2,000~3,000	4.03
3,000~5,000	3.84
5,000~10,000	5.46
>10,000	67.09

HWP-F, Infant formula containing hydrolyzed whey protein.

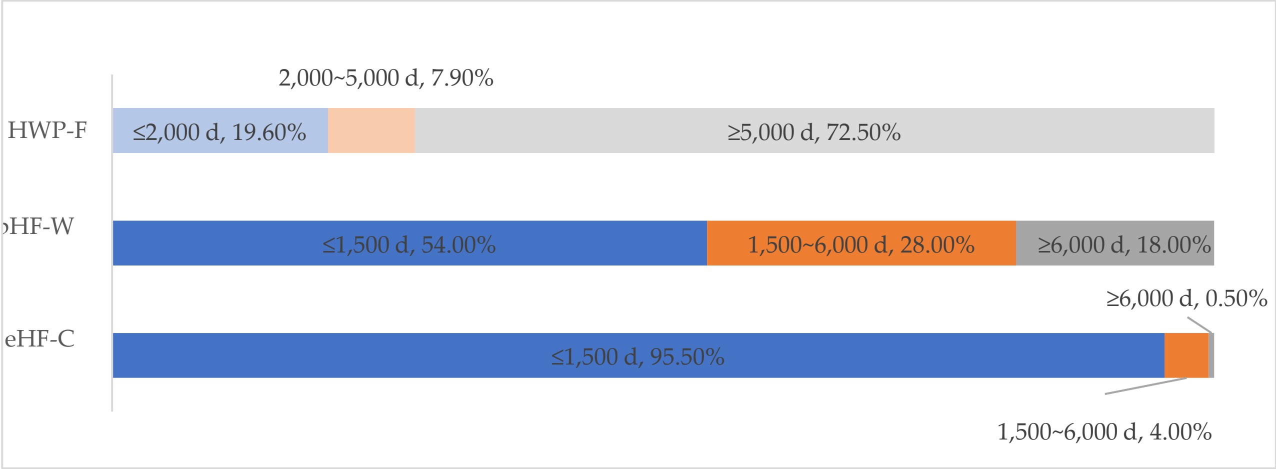

Figure S1. Comparation of Molecular weight distribution between HWP-F and commercial pHF-W and eHF-C. HWP-F, Infant formula containing hydrolyzed whey protein. pHF-W and eHF-C are well-established products [1].

Table S2. Weight, length, head circumference, and BMI and their z-scores at baseline, and 7 and 13 weeks of age of breast-, IWP-F-, and HWP-F-fed term infants (Per protocol analyzed).

	Baseline			***p*** [3]	7wk			***p*** [3]	13wk			***p*** [3]	***p*** [4]
	BF	IWP-F	HWP-F		BF	IWP-F	HWP-F		BF	IWP-F	HWP-F		
**Absolute values** [1]													
Weight, kg	3.6±0.5	3.5±0.4	3.6±0.5	0.707	5.3±0.5	5.3±0.6	5.2±0.7	0.763	6.5±0.7	6.5±0.6	6.5±0.8	0.926	0.475
Crown-heel Length, cm	51.3±2.1	51.3±2.0	51.7±2.4	0.505	57.4±2.1	57.5±2.2	57.6±2.4	0.894	61.5±2.1	61.7±2.2	61.6±2.8	0.853	0.591
Head circumference, cm	34.9±1.4	34.8±1.2	35.1±1.3	0.604	38.1±1.2	37.7±1.1	38.1±1.3	0.096	39.9±1.1	39.8±1.1	39.9±1.2	0.914	0.234
BMI, kg/m^2	13.5±1.4	13.4±1.2	13.4±1.4	0.907	16.1±1.3	15.9±1.2	15.7±1.6	0.371	17.2±1.5	17.0±1.5	17.1±1.6	0.889	0.699
***z*-scores** [1,2]													
Weight-for age *z*-score	0.20±0.89	0.20±0.76	0.28±0.97	0.836	0.40±0.68	0.42±0.81	0.16±1.05	0.205	0.49±0.73	0.48±0.74	0.35±0.93	0.547	0.310
Length-for-age *z*-score	0.31±1.11	0.36±1.10	0.52±1.25	0.562	0.42±0.98	0.57±1.04	0.45±1.07	0.720	0.46±0.96	0.54±1.08	0.34±1.23	0.627	0.246
Weight-for-length *z*-score	-0.35±1.20	-0.40±1.15	-0.42±1.20	0.951	0.15±1.08	-0.00±1.07	-0.07±1.08	0.546	0.28±1.03	0.22±1.02	0.22±1.26	0.940	0.832
Head circumference-for-age *z*-score	0.07±1.13	-0.00±1.00	0.15±1.06	0.738	0.00±0.98	-0.29±0.97	-0.13±1.13	0.321	-0.08±0.88	-0.15±0.92	-0.27±0.98	0.542	0.276
BMI-for-age *z*-score	0.03±1.02	-0.02±0.88	-0.02±1.07	0.953	0.23±0.88	0.15±0.90	0.02±0.96	0.464	0.33±0.96	0.26±0.93	0.22±1.13	0.840	0.941

BF, breast-fed; IWP-F, Infant formula containing intact whey protein; HWP-F, Infant formula containing hydrolyzed whey protein; BMI, body mass index; SD, standard deviation; IQR, interquartile range. [1] Values are means ± SDs. [2] *z*-scores were calculated by using the World Health Organisation (WHO) growth standards. [3] The *p* values were derived from one-way analysis of variance (ANOVA) model tests for the difference between the three groups. [4] The *p* values represent the time × treatment interactions, derived from repeated measures ANOVAs.

Table S3. Gains in weight, length, and head circumference of study infants (Per protocol analyzed).

	Group			p [2]	p [3]
	BF	IWP-F	HWP-F		
Weight, g/day [1]					
Baseline – 7 wk	40.0±10.2	41.6±11.8	37.6±14.7	0.215	0.085
Baseline – 13 wk	34.4±6.5	35.1±7.3	34.1±8.6	0.787	0.465
Crown-heel Length, cm/day [1]					
Baseline – 7 wk	0.14±0.05	0.15±0.05	0.13±0.05	0.137	0.157
Baseline – 13 wk	0.12±0.02	0.12±0.03	0.12±0.03	0.287	0.337
Head circumference, cm/day [1]					
Baseline – 7 wk	0.08±0.03	0.07±0.03	0.07±0.03	0.291	0.224
Baseline – 13 wk	0.06±0.01	0.06±0.02	0.06±0.02	0.629	0.576

BF, breast-fed; IWP-F, Infant formula containing intact whey protein; HWP-F, Infant formula containing hydrolyzed whey protein; [1] Values are means ± SDs; [2] The p values were derived from one-way analyses of variance; [3] The p values were derived from covariance analyses, adjusting for infant birth weight, gender, recruited center and growth indices at baseline.

Table S4. Feeding tolerance of study infants (Per protocol analyzed).

	Group			p [2]
	BF	IWP-F	HWP-F	
Number of daily feeding [1]				
2wk	9.7±1.9	8.7±1.8 [3]	8.6±1.8 [3]	0.002
7wk	9.4±1.8	8.4±1.8 [3]	8.4±1.6 [3]	0.002
13wk	8.8±1.8	7.7±1.7 [3]	7.8±1.7 [3]	0.002
Volume of formula intake, mL/kg/day [1]				
2wk	N/A	127.9±49.2	134.7±53.4	0.470
7wk	N/A	126.7±39.1	153.9±52.6	0.002
13wk	N/A	116.4±35.6	141.3±39.9	0.001
Number of daily crying [1]				
2wk	1.3±2.2	1.2±2.1	1.1±1.7	0.950
7wk	0.9±1.9	1.3±2.2	1.0±1.8	0.441
13wk	0.4±1.2	0.8±1.9	0.7±1.5	0.385

BF, breastfeeding; IWP-F, Infant formula containing intact whey protein; HWP-F, Infant formula containing hydrolyzed whey protein; N/A, not available; [1] Values are expressed as means ± SDs; [2] p values were derived from ANOVAs for normally-distributed data, and chi-square tests or Fisher's exact tests for categorical data; [3] Pairwise comparison was done using the Bonferroni test, a significantly difference from the BF group was indicated by $p < 0.05$.

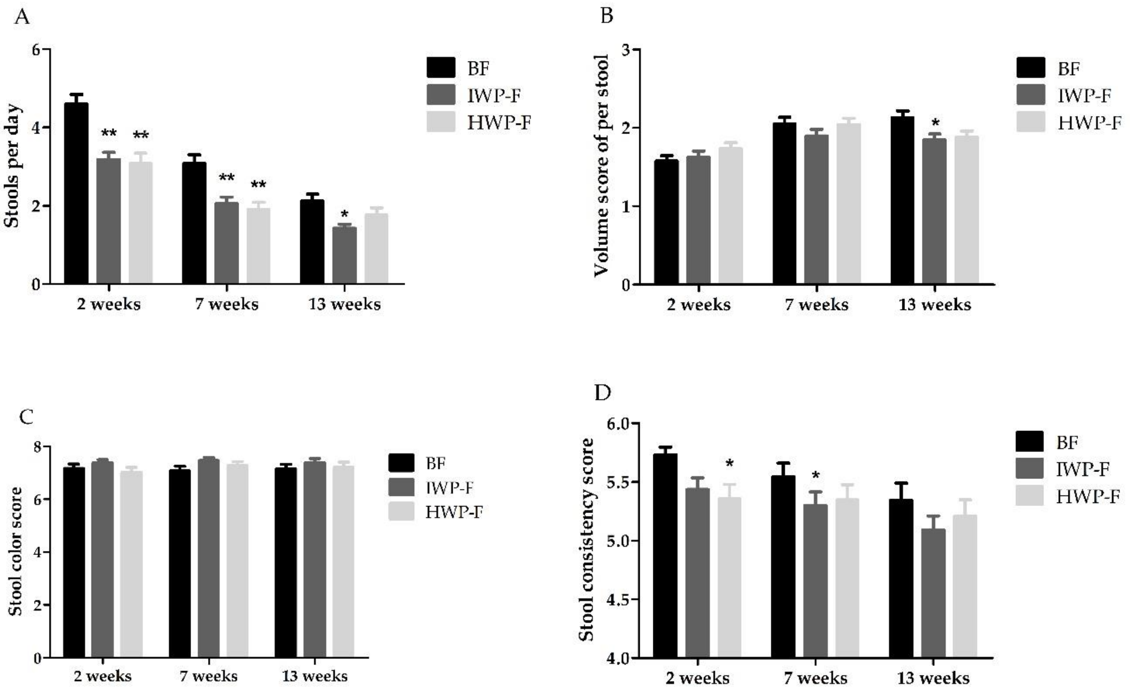

Figure S2. Stool characteristics (shown as mean and standard error) at 2, 7 and 13 weeks of age in breast-, IWP-F-, and HWP-F-fed term infants. (**A**) Stool frequency, indicated as stools per day. (**B**) Volume score per stool, expressed as stool diameter: 1 ≤7.5 cm; 2 >7.5 cm and <13.0 cm; 3 ≥13.0 cm. (**C**) Stool color score, calculated using the Infants Stool Color Chart, 1 indicates the most abnormal stool score, and 9 is the most normal. (**D**) Stool consistency score, rated based on the simplified Bristol Stool Form Chart, 1 = hard lumps, 2 = sausage-shaped but lumpy, 3 = formed but with cracks, 4 = smooth and soft, 5 = soft blobs with clear-cut edges, 6 = mushy. Thus, the range of possible scores is 0–6; a higher score is closer to a softer stool. Significant differences compared with the BF group are indicated as follows: **: $p < 0.001$; *: $p < 0.05$. HWP-F, Infant formula containing hydrolyzed whey protein; IWP-F, Infant formula containing intact whey protein; BF, breast-fed.

References:

1. Von Berg A.; Koletzko S.; Grubl A.; Filipiak-Pittroff B.; Wichmann H.E.; Bauer C.P.; Reinhardt D.; Berdel D. The effect of hydrolyzed cow's milk formula for allergy prevention in the first year of life: the German Infant Nutritional Intervention Study, a randomized double-blind trial. *J. Allergy Clin. Immunol*. **2003**, *111*, 533-540. [CrossRef] [PubMed]
